# A High Throughput Screen Identifies Chemical Modulators of the Laminin-Induced Clustering of Dystroglycan and Aquaporin-4 in Primary Astrocytes

**DOI:** 10.1371/journal.pone.0017559

**Published:** 2011-03-07

**Authors:** Geoffroy Noël, Sarah Stevenson, Hakima Moukhles

**Affiliations:** Department of Cellular and Physiological Sciences, University of British Columbia, Vancouver, Canada; National Institute on Aging Intramural Research Program, United States of America

## Abstract

**Background:**

Aquaporin-4 (AQP4) constitutes the principal water channel in the brain and is clusteredat the perivascular astrocyte endfeet. This specific distribution of AQP4 plays a major role in maintaining water homeostasis in the brain. A growing body of evidence points to a role ofthe dystroglycan complex and its interaction with perivascular laminin in the clusteringof AQP4 atperivascular astrocyte endfeet. Indeed, mice lacking components of this complex or in which laminin-dystroglycan interaction is disrupted show a delayed onset of brain edema due to a redistribution of AQP4 away from astrocyte endfeet. It is therefore important to identify inhibitory drugs of laminin-dependent AQP4 clustering which may prevent or reduce brain edema.

**Methodolgy/Principal Findings:**

In the present study we used primary rat astrocyte cultures toscreen a library of >3,500 chemicals and identified 6 drugs that inhibit the laminin-induced clustering of dystroglycan and AQP4. Detailed analysis of the inhibitory drug, chloranil, revealed that its inhibition of the clustering is due to the metalloproteinase-2-mediated ß-dystroglycan shedding and subsequent loss of laminin interaction with dystroglycan. Furthermore, chemical variants of chloranil induced a similar effect on ß-dystroglycan and this was prevented by the antioxidant N-acetylcysteine.

**Conclusion/Significance:**

These findings reveal the mechanism of action of chloranil in preventing the laminin-induced clustering of dystroglycan and AQP4 and validate the use of high-throughput screening as a tool to identify drugs that modulate AQP4 clustering and that could be tested in models of brain edema.

## Introduction

Cerebral edema with excess accumulation of water and cellular swelling is a common consequence of stroke, traumatic brain injury, brain tumor and meningitis. In the normal brain, water is distributed between cerebrospinal fluid, blood, intracellular and interstitial compartments and moves between these compartments in response to osmotic gradients. In pathological conditions, the abnormal accumulation of water in brain parenchyma gives rise to either cytotoxic or vasogenic edema [1]. Cytotoxic edema is seen in early cerebral ischemia and is primarily characterized by an increase in astrocyte volume due to enhanced water flux from the bloodstream into these cells. Vasogenic edema is often seen following brain tumor formation and is characterized by enhanced water entry in the interstitial compartment of the brain due to the disruption of the blood-brain barrier [1].

Aquaporin-4 (AQP4), the principal water channel in the brain, is mainly expressed at the interface between the brain tissue and the blood at the perivascular astrocyte endfeet [2]. Recent studies in the AQP4 knockout or overexpressing mice demonstrated a dual role for AQP4 in the pathophysiology of brain edema [1], [3], [4], [5], [6], [7]. Indeed, there is increasing evidence that AQP4 deficiency is associated with reduced water entry into the brain and reduced water outflow from the brain parenchyma in edema models that include water intoxication, focal cerebral ischemia, bacterial meningitis, cortical freeze-injury and brain tumor [1], [4]. Because AQP4 permits bidirectional water transport it has been shown that it plays a role not only in the early accumulation of water in cytotoxic edema [4] but also in the removal of excess water in vasogenic edema [1], [8], [9], [10]. Therefore, blocking AQP4 or inhibiting its clustering around blood vessels would reduce water entry which may be beneficial in preventing cytotoxic edema at early stages of stroke. On the contrary, activating AQP4 or increasing its clustering around blood vessels would promote the extrusion of the excess water from the brain preventing thereby vasogenic edema.

Several studies have focused their effort in designing drugs that specifically inhibit AQP4 function [11], [12], [13], [14], [15], [16], [17]. To date, only three drugs, tetraethylammonium (TEA), 6-ethoxybenzothiazole-2-sulfamide (EZA) and acetazolamide (AZA) have been identified as potential inhibitors of AQP4 activity. However, the ability of these candidate blockers to inhibit cell swelling upon hypo-osmotic chockhas been disputed [18]. In an attempt to identify other drugs that inhibit the function of AQP4 in water transport, Mola et al [15] developed a functional high-throughput assay based on the measurement of osmotically-induced cell volume changes to screen several libraries of drugs and identified four blockers of AQP4-mediated water transport.

Multiple lines of evidence suggest that the integrity ofthe dystroglycan complex (DGC) is essential for the properlocalization and function of AQP4. This multiprotein complex includes numerous intracellular proteins such as syntrophin, dystrobrevin and dystrophin downstream of dystroglycan (DG) [19], [20]. DG is post-translationally cleaved into a transmembrane protein, β-DG, and an extracellular protein, α-DG. Alpha-DG binds non-covalently to the short extracellular domain of ß-DG as well as to the extracellular matrix proteins laminin, agrin and perlecan as well as neurexin [21], [22], [23], [24]. Intracellularly, ß-DG binds directly to dystrophin, which in turn binds dystrobrevin and syntrophin. Interestingly, DG, dystrophin, dystrobrevin and syntrophin are co-clustered with AQP4 at perivascular astrocyte endfeet [25], [26]. We have shown in astrocyte cultures that this co-clusteringis mediated by the interaction of DG with extracellular laminin-1 [27], [28]. Likewise, we have demonstrated that α-DG interaction with perivascular laminin is key to the polarized distribution of AQP4 at astrocyte endfeet in brain [25]. Furthermore, the deletion of α-syntrophin or mutations of dystrophinresult in the loss of AQP4 clustering, relocating it away from astrocyte endfeet [29], [30], [31]. Most importantly, theloss of AQP4 polarized distribution in thesemice resulted in a beneficial effect as it delayed the onset of brain edema [32], [33]. Therefore, treatments using drugscapable of disrupting the clustered distribution of the DGC at astrocyte endfeet will ultimately leadto the disruption of AQP4 distributionas well.

In the present study we used a well established assay of laminin-induced clustering of the DGC and AQP4 in primary astrocyte cultures to screen a library of >3,500 chemicals and identified 5 enhancers and 6 inhibitorsof this clustering. Here, we focused our analysis on chloranil,one the inhibitory drugs, anddemonstrate that it causes cell surface cleavage of the 43 kDa ß-DGto a 31 kDa form via a metalloproteinase-mediated mechanism. Chemical variants of chloranil also induce ß-DG sheddingleading to the loss of cell surface tethering of α-DG andsubsequent severance of laminin binding,resulting in the inhibition of DG and AQP4 clustering. This study provides evidence that the DGC is a valuable target for the disruption of laminin-induced AQP4 clustering andvalidates the high-throughput screen on primary astrocyte cultures as a powerful tool in the discovery of potential therapeutic drugs for both cytotoxic and vasogenic edema that occur in brain disorders including stroke, tumour, hydrocephalus, infection and traumatic brain injury.

## Materials and Methods

### Chemicals

The 3,594 chemicals used in the screen were from the Pretswick, Sigma LOPAC, Microsource Spectrum and Biomol natural products collections, and were provided by the Canadian Chemical Biology Network (www.ccbn-rbc.ca). P-chloranil (referred to as chloranil in the rest of the text), benzoquinone and flunarizine were purchased from Sigma-Aldrich and Hoechst 33342 was from Invitrogen. Prinomastat (AG3340) was purchased from Agouron Pharmaceuticals, the tissue inhibitors of metalloproteinases TIMP1 and TIMP2 were a generous gift from Dr. C.M. Overall (University of British Columbia, Vancouver)and TIMP3was purchased from R&D Systems (Minneapolis, MN, USA). N-acetyl-L-cysteine (NAC) was purchased from Sigma-Aldrich.

### Antibodies

The following antibodies were used: rabbit anti-AQP4 raised against rat GST-AQP4 residues 249–323 (Alomone Laboratories, Jerusalem, Israel), mouse anti-ß-DG, 43DAG1/8D5, raised against 15 of the last 16 C-terminal amino acids of human dystroglycan (Novocastra Laboratories, Newcastle-upon-tyne, UK), mouse anti- syntrophin, SYN1351, raised against *Torpedo* syntrophin (Affinity Bioreagents, USA), and mouse anti-ß-actin raised against a mouse synthetic peptide (Sigma, USA).

### Chemical screen for modulators of laminin-induced dystroglycan clustering in astrocytes

Experiments were performed using postnatal day 1 Sprague-Dawley rats (Charles River) in accordance with protocols (#A06-0319)approved by the animal care committee of the University of British Columbia. Primary hippocampal astrocyte cultures were prepared from postnatal day 1Sprague-Dawley rats (Charles River). Hippocampi were dissected, and meninges and choroid plexus were removed. They were then cut into small pieces and incubated for 25 min with trypsin (3.0 mg/ml; Gibco, Burlington, Canada). Dissociated hippocampi were then plated in culture flasks and grown in Dulbecco's modified Eagle's medium (DMEM) supplemented with 10% fetal bovine serum, 1% penicillin-streptomycin and 1 mM L-glutamine (Gibco) for 2–3 weeks. To remove microglia and oligodendrocyte progenitors, the flasks were shaken the day following the plating.

The culture medium was changed every 3 days. After trypsinization, the cells were plated in PerkinElmer View 96-well plates at 8000 cells per well. One day after plating, the cells were treated for 7 h with 20 nM Engelbreth-Holm-Swarm Sarcoma laminin-1 (Sigma-Aldrich), washed with warn phosphate buffered saline (PBS) and fixed with 4% (w/v) paraformaldehyde in 0.1 M phosphate buffer for 20 min followed by rinsing in PBS, 3×15 min. Four hours before fixation, chemicals were transferred from stock plates (5 mM in DMSO) to each well for a final concentration of ≈15 µM using a Biorobotics Biogrid II robot equipped with a 0.7 mm diameter 96-pin tool that adds∼20 nl per well. Fixed cells were incubated for 1 h at room temperature (20–22°C) in a solution containing 2% bovine serum albumin (Sigma) and 0.25% Triton X-100. Immunolabelling was performed by incubating the cells at room temperature for 1 h in the presence of the primary antibody against ß-DG (1/100). Subsequently, cells were rinsed with PBS (3×15 min) and incubated with Alexa Fluor 568 goat anti-mouse IgG (1/200; Molecular Probes, USA). After several washes with PBS, the cells were incubated with PBS containing 500 ng/ml Hoechst 33342 for 15 min at room temperature. Plates were read in a Cellomics™ Arrayscan V^TI^ automated fluorescence imager. Cells were photographed using a 20× objective in the Hoechst and TRITC(XF-100 filter) channels. The compartment analysis algorithm was used to identify the nuclei, apply a cytoplasmic mask and quantitate TRITC spotsin the TRITC channel fixed at 250 pixel intensity units. Fluorescence intensity in the TRITC channel was gated at 10 average pixel intensity units inside the cytoplasmic mask to select against diffuse ß-DG staining. The total pixel intensity for clustered ß-DG was acquired as ‘circ spot total intensity ch2’. Compounds inducing an increase or decrease greater than 1.5 in clustered ß-DG staining were considered as active. Wells showing 3-fold or higher increase in clustered ß-DG staining over control were re-examined to eliminate any false positives resulting from precipitation of fluorescent compounds. Similarly, compounds causing a decrease in cell number wereconsidered as toxic chemicalsand disregarded. The Z-factor of the assay was determined from clustered ß-DG measured in cells treated with DMSO (negative control) or laminin (positive control). Effective drugs identified in the screening assay were further evaluated in a dose-response curve where DMSO concentration was kept at 0.1% in all wells for all experiments.

### Cell viability assay

Cells were seeded in 96-well plates at 8000 cells per well and grown for 24 h prior to the treatment with the drugs for 4 h as described above. The drugs and media were then removed and cell viability was measured using the 3-[4,5-dimethylthiazol-2-yl]-2.5-diphenyl-tetrazolium bromide (MTT) assay (Sigma, USA). Cells were incubated for 2 h at 37°C with MTT, then 20% sodium dodecyl sulfate was added and the absorbance at 570 nm was measured after overnight incubation.

### Immunoblotting

Treated astrocytes were harvested and suspended in 0.5 ml of ice-cold extraction buffer (25 mM Tris pH 7.4, 25 mM glycine and 150 mM NaCl) containing 1% Triton X-100, 1× complete protease inhibitor cocktail and 5 mM EDTA. After 20 min incubation on ice, the lysates were centrifuged at 800 g for 10 min. Supernatant was collected and the protein concentration was quantified using a BCA protein assay kit (Pierce, USA). Extracted proteins were denatured by boiling for 4 min in reducing sample buffer and then loaded on 10% sodium dodecyl sulfate-polyacrylamide electrophoresis gels. The gels were electrotransferred to nitrocellulose membranes (Bio-Rad, Mississauga, ON, Canada) and the blots were probed with antibodies to ß-DG (1/300), AQP4 (1/1000), syntrophin (1/1000) andß-actin (1/10000). Bound antibodies were detected using horseradish peroxidase-conjugated goat anti-rabbit or goat anti-mouse IgGs (1/2000; Jackson ImmunoResearch, USA). Signals were visualized on Bioflex econo films (Interscience, Markham, ON, Canada) using chemiluminescence (Amersham Biosciences, Buckinghamshire, UK).

### Gelatin zymography

Media from treated astrocytes were collected and 10 µl aliquots per well were applied to 10% SDS-PAGE gels co-polymerized with 1% gelatin (Fisher, USA). After electrophoresis under non-reducing conditions, the gels were washed in 2.5% Triton X-100 for 1 h to remove SDS and then incubated for 16 h at 37°C in 20 mM Tris, 150 mM NaCl, 5 mM CaCl_2_ and stained with SimplyBlue™ Safestain (Invitrogen, USA). For the standards, we used the pre-stained protein ladder from Bio-Rad as well as purified gelatinase A (MMP-2) and B (MMP-9). Regions of gelatinolytic activity regions were observed as clear bands against a blue background.

## Results

### Development of an automated microscopy screenfor chemical modulators of the laminin-induced dystroglycan clustering in primary astrocyte cultures

**Figure 1:**
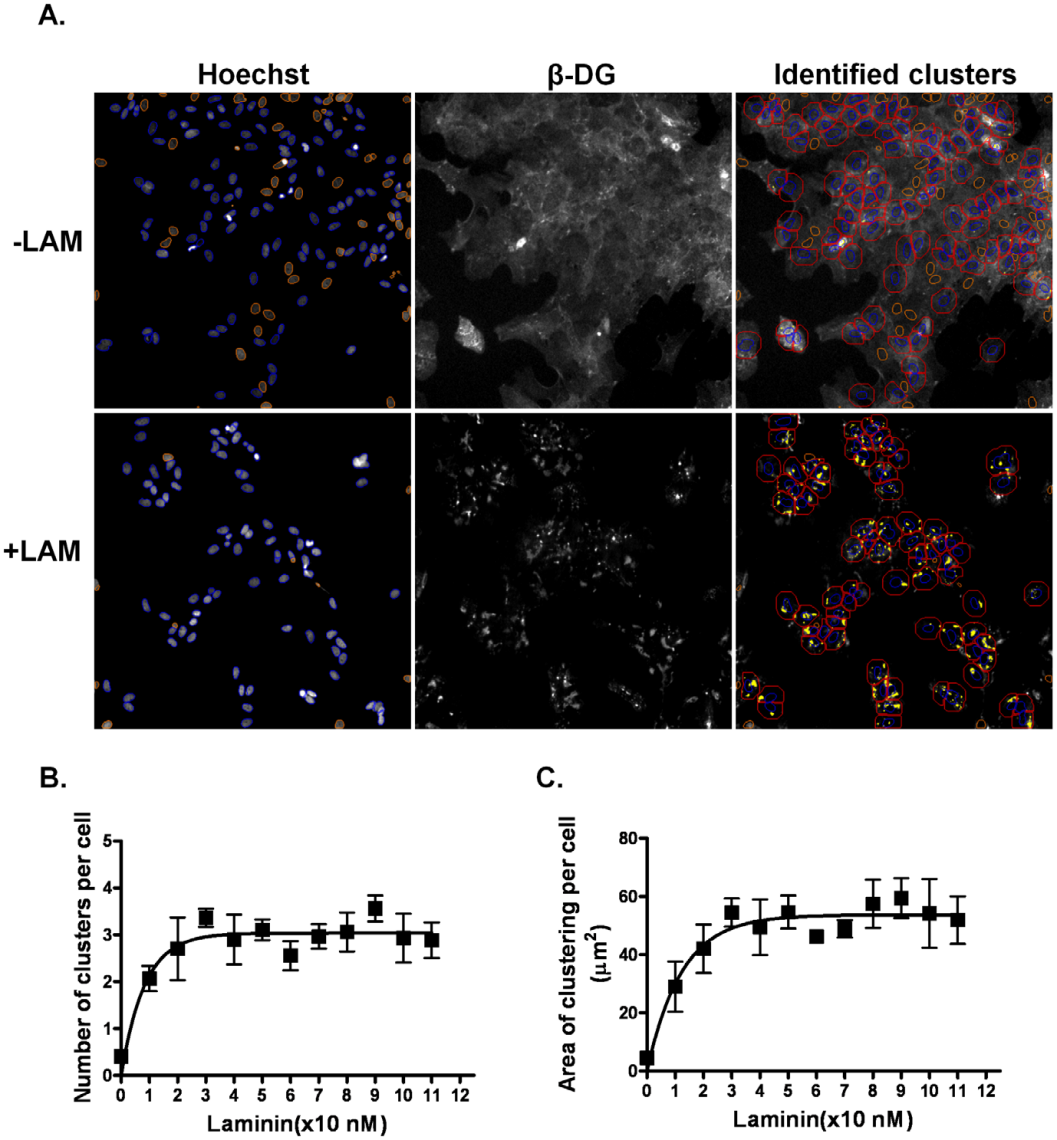
Validation of the Cellomics™ Arrayscan V^TI^ automated fluorescence imaging of the laminin-induced clustering of ß-dystroglycan in primary astrocyte cultures. A. Images of untreated astrocytes and astrocytes treated with 20 nM laminin-1 were acquired on the ArrayScan V^TI^ HCS Reader. The right panels are merged images of Hoechst 33342 (left panels) and ß-DG labelled astrocytes (middle panels). Automatic identification of the cytoplamic maskof individual astrocytes(red) and detected spots (yellow) by the Compartmental Analysis BioApplication. B, C. Dose-response curve of laminin-induced ß-DG clusteringin astrocytes grown in 96 well-plates. Each point represents the mean number of clusters or areas of clusters ± S.E.M. from three different experiments. Scale bar, 30 µm.

To screen libraries of compounds for modulators of the laminin-induced clustering of ß-DG in primary astrocyte cultures, we used an automated microscopy assay. The high-content screening instrument programmed to detect and quantify fluorescent spots enabled the automatic imaging of ß-DG fluorescent clusters on a Cellomics ArrayScan V^TI^ HCS Reader and analysis by the Compartmental Analysis BioApplication. Representative images obtained with this automated image analysis system are shown in Figure 1A in which each cellis outlined in red and nuclei of each live cell overlaid in blue. Individual clusters associated with each cell are shown in yellow (Fig. 1A). To determine the most suitable conditions for optimum detection of ß-DG clustering in astrocytes cultured in 96 well plates using the Cellomics reader, wetreated these cultures with increasing concentrations of laminin-1for 7 h. In the absence of laminin-1, ß-DG labelling was largely diffuse at the cell surface (Fig. 1A), however in the presence of laminina dose-dependent response both in the number and area of the clusters was observed reaching a plateau at 20 nM of laminin-1as previously reported in Muller glial cells (Fig. 1A–C; 21).

#### Evaluating assay performance

The values from untreated negative control and treated (20 nM laminin) positive control wells were used to assess the Z′ factor for the number of clusters and the total area covered by the clusters per cell. The values for the number of clusters (0.54) and the total area of clustering per cell (0.62), are greater than 0.5, indicating that thesecriteriaare appropriate for being used in this screening assay (Table 1). As demonstrated by the automated assay, the addition of laminin-1 for 7 h caused a 9.11 to 18.35-fold increase in the number and area of ß-DG clusters, respectively (Table 1). Other criteria shown in the Table 1 include the assay's signal/noise and signal window, both of which are greater than 2-fold, further demonstrating the robustness of this assay [34].

**Table 1:** Evaluation of assay performance.

	Number of clusters per cell	Area of clustering per cell (µm^2^)
***Z′ factor***	0.54302	0.62
***Signal to background***	9.115798017	18.35
***Signal to noise***	28.62252524	35.39
***Signal window***	4.625771126	6.42

ß-dystroglycan clustering is a suitable assay for detection using the Cellomics™ Arrayscan V^TI^ automated fluorescence imager. ß-DG clustering in laminin-treated astrocytes was used as the positive control and the spontaneous clustering occurring in the absence of exogenous laminin was used as the negative control. Z′ = 1−((3σ_max_+3σ_min_)/|μ_max_−μ_min_|), where μ_max_ is the mean positive control signal, σ_max_ is the standard deviation of the positive control signal, μ_min_ is the mean negative control signal, and σ_min_ is the standard deviation of the negative control signal. Signal/background (fold increase) = μ_max_/μ_min_. Signal/noise = (μ_max_−μ_min_)/σ_min_. Signal window = (μ_max_−μ_min_−3(σ_max_+σ_min_))/σ_max_ [34].

#### Screening of compound collections

**Figure 2:**
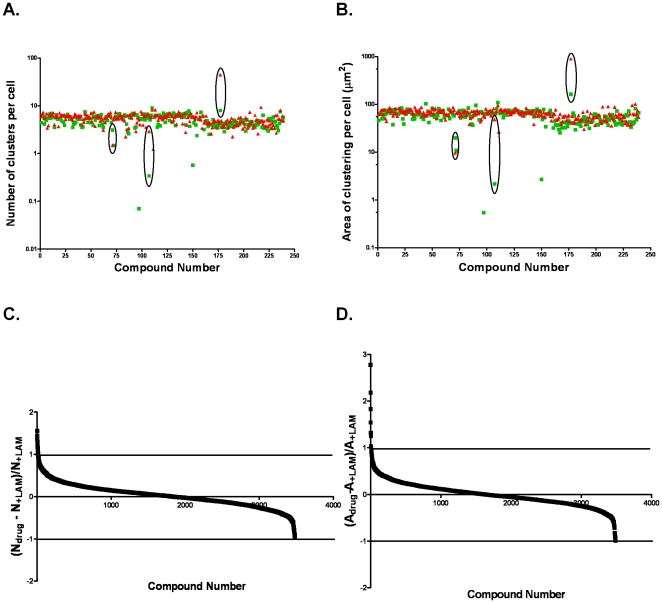
Identification of modulators of ß-dystroglycan clustering using a high throughput screen of a library containing 3,594 drugs. A, B. Duplicate experiments (red and green)from three 96-well plates corresponding to approximately 200 drugs are represented. Drugs inducing a change greater than 1.6 fold in ß-DG clustering in laminin-treated compared to untreated astrocytes in both experiments (as circled in black) were considered effective and used for subsequent testing. C, D. Normalized fold differences in the number and area of the clusters plotted for each of the 3,594 compounds tested.

**Figure 3:**
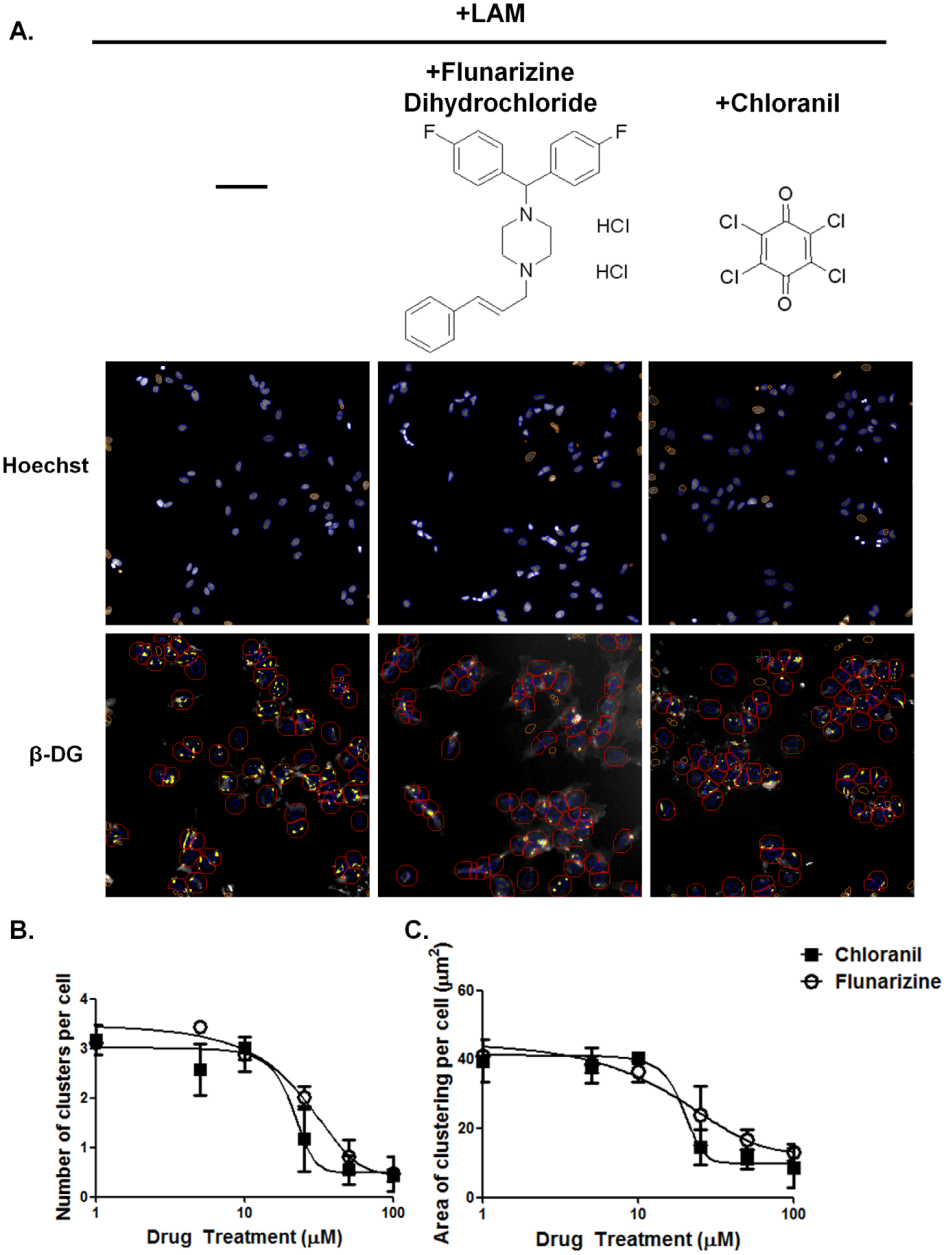
Dystroglycan clustering in cells treated chloranil and flunarizine dihydrochloride. **A.** Primary astrocytes were treated for 7 h with 20 nM laminin-1alone, 20 nM laminin-1 and 15 µM chloranil or 20 nM laminin-1 and15 µM flunarizine during the last 4 h. The chemical structures of chloranil and flunarizine are represented. B, C. Dose-response curve of laminin-induced ß-DG clustering in astrocytes treated with increasing concentrations of chloranil or flunarizine (2.5 to 100 µM). Clustered staining was automatically quantified using the Cellomics ArrayScan V^TI^ HCS Reader. Scale bar, 30 µm.

A collection of 3,584 drugs and pharmaceutically active chemicals was tested at a concentration of 15 µM for the final 4 h of the 7 h laminin incubation. In addition to automatically reporting the number of clusters and their total area, the number of cells (i.e., number of nuclei) and the nuclear area were simultaneously measured. Chemicals causing 80% reduction in cell number were considered cytotoxic and were eliminated from our analysis. Compounds causing over 1.6-fold increase or decrease in the clustering ofß-DG without causing cell death were considered as active (Fig. 2 C and D). Examples of these compounds are illustrated in Figure 2A and Bin which data compiled from three 96-well plates corresponding to approximately 200 compounds are shown. Twelve active chemicals were initially identified as inhibitors of the clustering,but subsequent testing by dose-response proved that only 6 of these were true inhibitors inducing a reduction of ß-DG clustering ranging from 2.2 to 17.1-fold (Fig. 3 and data not shown). To our knowledge, none of these 6 compounds have been previously reported to reduce laminin-DG interaction or laminin-induced clustering of the DGC.

Subsequent analysis has shown that two of the identified inhibitors, namely chloranil and flunarizine, induce a dose-dependent decrease in ß-DG clustering (Fig. 3B). To determine the most efficient concentration of chloranil and flunarizine required to induce a 50% inhibition (EC_50_) of laminin-induced clustering, astrocytes were incubated with 20 nM laminin-1 and either chloranil of flunarizine at concentrations ranging from 1 to 100 µM (Fig. 3B). Thedata from thisdose-response experimentare represented as curves that were fitted using a sigmoidal function allowing the determination of the EC_50_. Figure 3B shows a dose-dependent inhibition of the laminin-induced ß-DG clustering by both drugs, however chloranil presents an EC_50_ (∼20 µM) lower than that of flunarizine (∼30 nM) indicating that chloranil is more potent than flunarizine.

### Characterization of the effect of chloranil and flunarizine on ß-dystroglycan and AQP4 clustering

**Figure 4:**
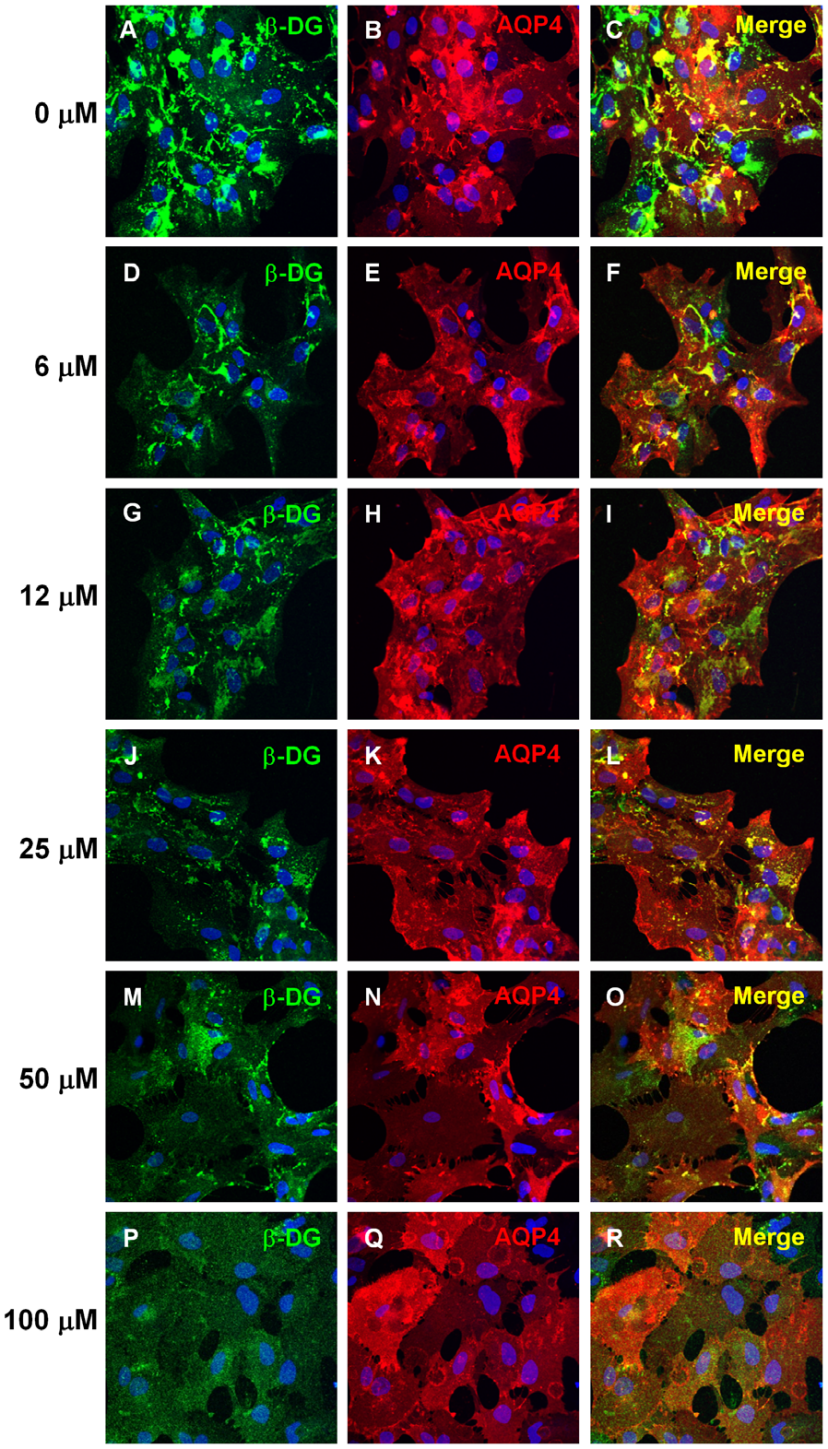
Effect of chloranil on the laminin-induced co-clustering of ß-DG and AQP4. Primary astrocytes were treated for 7 h with 20 nM laminin and15 µM chloranil during the last 4 h. The concentration of chloranil varied from 0 (A,B,C), 6(D,E,F), 12(G,H,I), 25 (J,K,L), 50 (M,N, O) to 100 µM (P,Q,R). The cells were fixed and labelled for μ-DG (A, D, G, J, M and P) and AQP4 (B, E, H, K, N and Q). Clustered staining was quantified using confocal microscopy. Scale bar, 30 µm.

To assess whether AQP4 clustering is also reduced in the presence of chloranil and flunarizine, ß-DG and AQP4 clusters were examined at higher resolution by laser confocal microscopy. The data show that chloranilsignificantly inhibits the laminin-induced AQP4 clustering and as for ß-DG, this inhibition is dose-dependent (Fig. 4). Similar results were found for flunarizine (data not shown).

**Figure 5:**
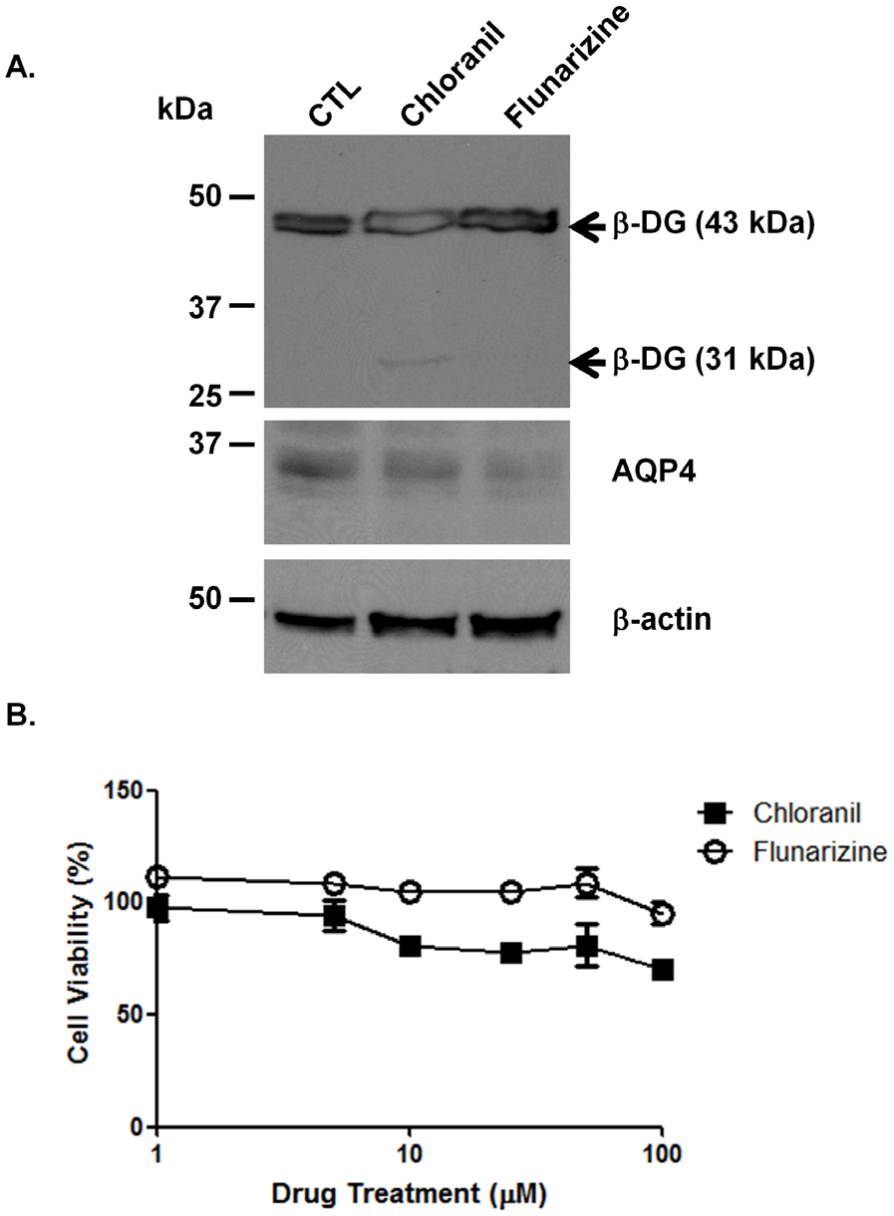
Effect of chloranil and flunarizine on astrocyte survival and ß-dystroglycan, and AQP4 expression. A. Primary astrocytes were incubated for 4 h with 15 µM of active chemicals. Extracted proteins were loaded (30 µg/lane) and analyzed for ß-DG, syntrophin and AQP4 expression levels by western blot analysis. Note the 31 kDa band under the 42 kDa band corresponding to the cleaved form of ß-dystroglycan upon chloranil treatment. B. Primary astrocytes were incubated for 4 h with different concentrations of the active chemicals. Chemicals and media were washed away and the cells were assayed for cell viability by MTT assay.

To ensure that the reduction in ß-DG and AQP4 clustering was not due to either cytotoxicity or reduction in ß-DG or AQP4 expression levels, we assessed cell viability using the MTT assay andß-DG and AQP4levels by immunoblotting. Figure 5 shows that while ß-DGexpression level remains unchanged in the presence of flunarizine that ofAQP4is slightly decreased. Interestingly, chloranil causes the cell surface cleavage of full length 43 kDa ß-DG and consequent formation of the 31 kDa fragment of ß-DG (Fig. 5A). The size of this fragment is consistent with the cleavage of the short extracellular domain of ß-DG that has been reported to be mediated by metalloproteinases. We next investigated the effectof increasing concentrations of chloranil and flunarizine on astrocyte survival. Four hours following the treatment with chloranil and flunarizine, we subjected the astrocytes to the MTT cell viability assay and found that chloranil caused a slight reduction in the total number of actrocytes but only at a very high concentration (100 µM; Fig. 5B). These results demonstrate that the inhibition of ß-DG and AQP4 clustering observed with <100 µM chloranil is not due to adverse effects on cell survival (Fig. 3 and 4).

### Chloranil-induced ß-dystroglycan shedding is mediated by metalloproteinases

**Figure 6:**
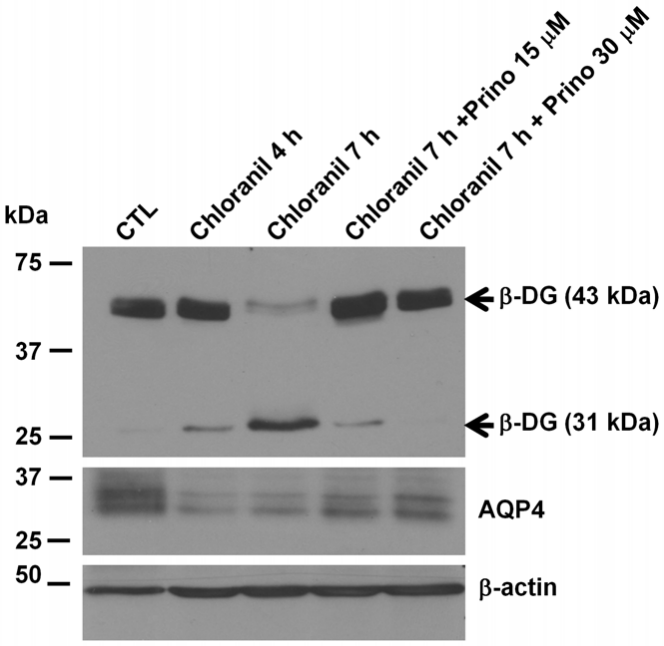
Time-dependent shedding of ß-dystroglycan by chloranil is blocked by the metalloproteinase inhibitor, prinomastat. Primary astrocytes were incubated with 25 µM chloranil for 4 or 7 h with or without addition of 15 nM or 30 nM prinomastat. Extracted proteins were loaded (30 µg/lane) and analyzed for ß-DG and AQP4 expression levels by western blot analysis. Note the increase in signal intensity of the 31 kDaß-DG band after extended incubation with chloranil and its disappearance with prinomastat co-incubation.

Since the formation of the 31 kDa ß-DG fragment in chloranil-treated astrocytes is consistent with a proteolytic cleavage of ß-DG possibly implicating metalloproteinases, we tested whether longer exposure to chloranil results in increased accumulation of the 31 kDaß-DG. The data show a more intense 31 kDa band and weaker 43 kDa band at 7 h compared to 4 h post-treatment (Fig. 6). This time-dependent accumulation of the proteolytic 31 kDaß-DG fragmentisconsistent with proteolytic activity. Increasing accumulation of the 31 kDa ß-DG fragment was also seen withincreased chloranil concentrationsranging from 15 µM to 50 µM(data not shown).

We next evaluated the role of metalloproteinases in the chloranil-induced ß-DG cleavage using the metalloproteinaseinhibitor, Prinomastat at 15 and 30 µM. Immunoblots of protein extracts of astrocytes co-incubated with 15 µM chloranil and15 or 30 µM prinomastat for 7 h show that prinomastat inhibits the chloranil-induced ß-DG cleavage partially when applied at 15 µM and completely at 30 µM (Fig. 6). These data show conclusively that the chloranil-induced cleavage of ß-DG is mediated by metalloproteinases.

**Figure 7:**
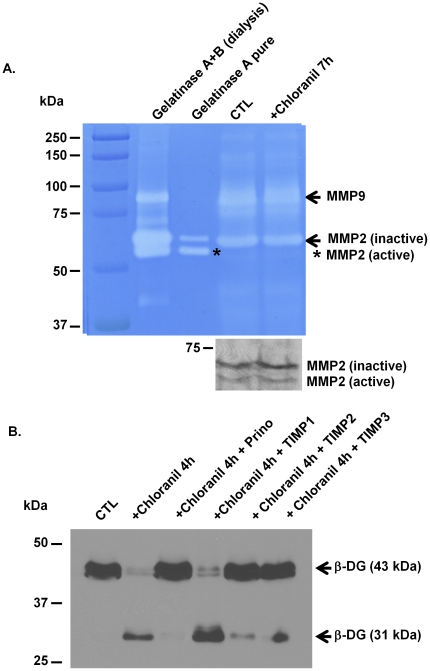
Gelatin zymography and the effect of tissue inhibitors of metalloproteinaseson the chloranil-induced shedding of dystroglycan. A. Primary astrocytes were incubated in the absence or the presenceof 25 µM chloranil for 7 h. Media samples were collected and analyzed by SDS-PAGE gelatin zymography. Note the clear bands against the blue background with an apparent molecular weight of ∼92 and ∼72 kDa, representing gelatinolytic activity of MMP-9 and MMP-2, respectively. The chloranil does not modulate the amount of MMP-2 and 9 or their activation state. B. Primary astrocytes were incubated for 4 h with 25 µM chloranil alone, 25 µM chloranil with 30 µMprinomastat, 25 µM chloranil with 110 nM TIMP1, 25 µM chloranil with 100 µM TIMP2 or 25 µM chloranil with 100 µM TIMP3. Protein extracts were loaded (30 µg/lane) and immunoblotted for ß-DG. Note that the chloranil-mediated shedding of ß-DG is inhibited by prinomastat, TIMP2and TIMP3 but not TIMP1.

Shedding of ß-DG at the cell surface has been studied in many cell types including tumor cells and glial cells. Several studies have reported that the two metalloproteinases, MMP-2 and MMP-9 areinvolved in this processin brain [35], [36]. To determine whether chloranil-induced shedding of ß-DG in astrocytes is mediated by these twometalloproteinases, we investigated their activity via gelatin zymography. Culture media from untreated astrocytes and chloranil-treated astrocytes were collected and analyzed by gelatin zymography. As standards, we used a mix of gelatinase A (MMP-2) and B (MMP-9) from CHO cells or purified gelatinase A. The gelatinolytic regions observed with both untreated and chloranil-treated astrocytes' culture media correspond to MMP-9 and the inactive form of MMP-2, as revealed by the clear bands at ∼92 and ∼72 kDa, respectively (Fig. 7A). No difference in intensity or activation state of either one of these MMPs was observed between the untreated and chloranil-treated astrocytes (Fig. 7A). Further analysis was conducted using the tissue inhibitors of metalloproteinases, TIMP1 (110 nM), and TIMP2(100 µM) that inhibit selectively MMP-2 and MMP-9 but not the ADAMs (a disintegrin and a metalloproteinase) as well as TIMP3 (100 µM) that has a broader inhibition profile which includes MMPs, the tumor necrosis factor α-converting enzyme (TACE or ADAM17) and the related enzyme ADAM10 [37]. Astrocytes treated for 4 h with chloranil alone, chloranil with prinomastat or chloranil with TIMP1, TIMP2 or TIMP3 were analyzed by immunoblotting. Unlike prinomastat, TIMP1 did not inhibit the chloranil-induced shedding of ß-DG (Fig. 7B). However, both TIMP2 and TIMP3 were able to inhibit this effect (Fig. 7B).

### ß-dystroglycan sheddingby chloranil and its chemical variants is mediated by reactive oxygen species

**Figure 8:**
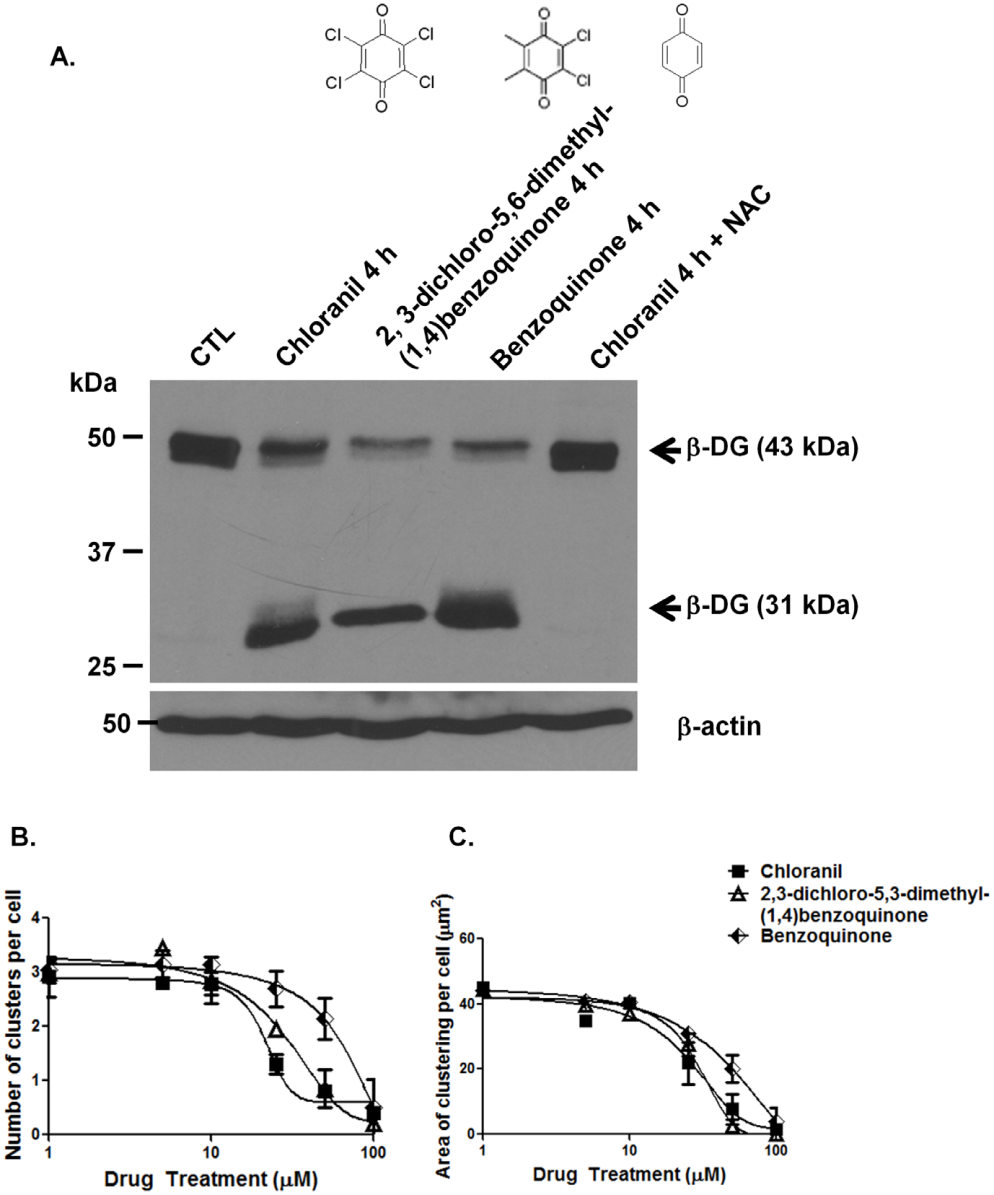
Effect of chloranil, 2,3-dichloro-5,6-dimethyl-(1,4)benzoquinone and benzoquinone on ß-dystroglycan shedding and laminin-mediated clustering. A. Primary astrocytes were incubated for 4 h with 15 µM of active chemicals. Protein extracts were loaded (30 µg/lane) and analyzed for ß-DG expression levels by western blot analysis. Note the 31 kDa band corresponding to the cleaved form of ß-DG upon chloranil as well as 2,3-dichloro-5,6-dimethyl-(1,4)benzoquinone or benzoquinone treatment. The chloranil induced shedding of ß-dystroglycan is prevented by co-incubation of the cells with 15 µM of chloranil and 1 mM of the ROS scavenger, N-Acetylcysteine (NAC). B, C. Primary astrocytes were treated for 7 h with 20 nM laminin and chloranil, 2,3-dichloro-5,6-dimethyl-(1,4)benzoquinone or benzoquinone during the last 4 h. The concentration of the compounds varied from 2.5 to 100 µM. Clustered staining was quantified using the automated microscopy assay.

Chloranil is a general electron acceptor that has a role in radical ion formation and has been shown to be most effective at producingreactive oxygen species (ROS) by accepting electrons from oxygen [38]. Based on this property of chloranil, its reported inability to interact or regulate collagenase activity [39] and the fact that metalloproteinases can be activated by ROS, we hypothesized that choranil inducesß-DG shedding by producing ROS that in turn activate specific metalloproteinases. To test this hypothesis,astrocytes were incubated either with chloranil alone or chloranil plus the ROS scavenger, N-acetyl-cysteine (NAC). Interestingly, NAC completely inhibited the proteolytic cleavage of ß-DG by chloranil (Fig. 8A).

Similar to chloranil,chemical variants of chloranil such as 2,3-dichloro-5,6-dimethyl (1,4) benzoquinoneand benzoquinone, induced ß-DG cell surface cleavage and a dose-dependent decrease both in the number and area of ß-DG clusters as assessedby immunoblotting (Fig. 8A) and immunofluorescence analyses (Fig. 8B). The effect of chloranil and its variants is most likely mediated by the benzoquinone which is common to these three aromatic compounds. The observation that these variants induce a similar effect to chloranil and that chloranil acts via the production of ROS suggest strongly that chloranil variants increase ROS production as well leading to the activation of metalloproteinases.

## Discussion

Several studies have supported the role of the DGC and its interaction with extracellular laminin in the polarized distribution of AQP4 at astrocyte endfeet abutting blood vessels. Indeed, the severance of the DG interaction with laminin in the DG-glycosylation deficient Large^myd^mouse, results in the loss of AQP4 localization at astrocyte endfeet without affecting its expressionlevel [25]. We have also shown that the laminin-induced clustering of AQP4 in astrocyte cultures requires DG [27], [28]. In addition, the deletion of α-syntrophin or mutations in the dystrophin gene result in themislocalization of AQP4, distributing it away from astrocyte endfeet [29], [30], [31]. It is noteworthy that AQP4 mislocalization in these mice results in a better outcome as it delays the onset of brain edema [32], [40]. A study in the AQP4-null mouse reported brain swelling in models of vasogenic edema where excess fluid accumulates in the extracellular space, due to impairment of the AQP4-dependent water clearance [41]. On the contrary, another study in the AQP4 null mouse reported reduced brain swelling and improved neurological outcome following water intoxication and focal cerebral ischemia [4]. Thisdefines a role for AQP4 in the development of cytotoxic (cellular) cerebral edema where excess fluid accumulates mainly in the astrocyte foot processes [4]. Modulators of AQP4 function, expression or perivascularclusteringmay therefore have a therapeutic application to reduce cytotoxic edema by blocking water influx in astrocytes at early stages of stroke. Indeed, recent studies have identified potential inhibitors of AQP4-mediated watertransport *in vitro* [12], [14], [15], however the data by Huber et al [12], [14] have been disputed [18].

In the present study we implemented a high-throughput screen in primary astrocyte cultures toidentifycompounds that modulate the laminin-induced clustering of ß-DG and AQP4. First, we showed that the CellomicsTMArrayscan VTI automated fluorescence imaging is suitable for efficient detection of the laminin-induced clustering of ß-DG (Fig. 1). Indeed, the Z′ factor (0.54),signal/background,signal/noise and signal window valuesdemonstrate that the number and area of the clusters are suitable parameters for the Cellomics-based screening (Table 1). Screening of the Pretswick, Sigma LOPAC, Microsource Spectrum and Biomol natural products collections identified a number of chemically active molecules, 6 of which inhibited ß-DG clustering. Here, we focused our study onflunarizine and chloranil, two inhibitors of the clustering, and show based on the EC_50_ that chloranil (EC_50_∼20 µM) is more potent than flunarizine (EC_50_∼30 µM). The effect of these drugs on ß-DG clustering was reversible (data not shown) and neither of them compromised astrocyte viability at the concentration used throughout the experiments. These are generally considered favourable properties for drugs with a therapeutic potential.

However, studies have reported that chloranil is able to generate DNA single-strand brakes in V79 chinese Hamster cells [42] and various levels of toxicity depending on the cell type [38], [43]. Indeed, amongst several quinones tested, chloranil was the only one to induce a weak toxicity in platelets [43] and was less toxic in PC12 cells than in primary hepatocytes [38]. We noted minimal toxicity on primary astrocytes at doses that induced a significant inhibition on ß-DG and AQP4 clustering, suggesting that the degree of chloranil toxicity is cell type-dependent.

Flunarizine plays the role of a calcium channel blocker in astrocytes [44], [45] and protects rats from severe ischemic cell death [46]. Its role on brain edema has been investigated and despite a remarkable recovery of the electroencephalographic activity in a rat model of cerebral ischemia [47] and an increased recovery rate of the electrical potential in a cat model of spinal contusion injury [48], flunarizine did not induce a significant inhibition of brain swelling. Furthermore, flunarizine has proven to be effective in preventing ischemia in subarachnoid hemmorrhage, although its effect on cerebral infarction has not been proven yet [49]. Further *in vivo* experiments aiming at investigating the role of flunarizine in models of cytotoxic brain edema addressing particularly its effect on DG and AQP4 perivascular distribution are warranted.

Chloranil is commonly used as an oxidizing agent in organic syntheses. Here we show that it induces the shedding ofß-DG ectodomain resulting in the formation of the 31 kDa ß-DG fragment and a similar effect isseenwith other oxidizing agents such as 2,3-dichloro-5,6-dimethyl-(1,4)benzoquinoneand benzoquinone, suggesting that the effect of chloranil and its variants on ß-DG is due toROS production. This is further substantiated by the observation that chloranil-induced ß-DG shedding is prevented by the anti-oxidant, NAC (Fig. 8). ß-DG shedding is typical of a metalloproteinase-mediated cell surface cleavage of ß-DG as previously reported [36], [50], [51]. Indeed, our data show that prinomastat, an inhibitor with selectivity for MMP-2, 9, 13, and 14,completely blocks the chloranil-induced cell surface shedding of ß-DG (Fig. 7B). These findings together with the established role of ROS in MMP activation [52], [53], [54] indicate that chloranil induces the production of ROS that in turn activates metalloproteinases leading to the cleavage of ß-DG ectodomain. This is in accordance with the report that cerebral ischemia seen after oxygen-glucose deprivation leads to a metalloproteolytic cleavage of ß-DG in astrocytes [55] and that prinomastat protects against ischemic brain injury as shown in hippocampal organotypic slices [56].

A number of studies have shown that ß-DG is a substrate for MMP-2 and 9 but not MMP-3, 8 or 13 [36], [50], [57], [58], [59]. Our data showing a lack of inhibition of the chloranil effect by TIMP1 argue against an MMP-9-mediated ß-DG shedding (Fig. 7B). However, the fact that both TIMP2 and 3 inhibit the chloranil-induced ß-DG shedding indicate that MMP-2 is one of the metalloproteinases targeted by chloranil. Although TIMP3 has the ability to inhibit not only gelatinases including MMP-2 but also the tumor necrosis factor-α (TNF-α)-containing enzyme (TACE or ADAM17) and the related enzyme ADAM10 [60], ADAM17 and 10 are unlikely targets of chloranil since prinomastat, which is a potent inhibitor of MMP-2 [61] but not ADAMs can completely inhibit the chloranil-induced ß-DG shedding. This, together with the TIMP2 inhibition, supports further the implication of MMP-2 in the chloranil-induced effect. It is noteworthy that the pro-MMP-2 (inactive MMP2; Fig. 7A) but not the active MMP-2 is detected in the culture medium harvested from astrocytes treated with chloranil, as reflected by the gelatine zymography assay. It is therefore possible that the activation of MMP-2 by chloranil occurs at or near the cell membrane where membrane-type MMPs (MT-MMPs) play a role in tethering MMP activity [62], which would explain the absence of active MMP2 in the culture medium. This suggests that TIMP2 and TIMP3 inhibition of MMP-2 activation by chloranil occurs at the cell surface. Indeed,membrane-bound pro-MMP2 processing is inhibited in the presence of high concentrations of TIMP2 [63] and TIMP3 [64].

In light of the fact that ROS-induced activation of MMPs is involved in blood brain barrier (BBB) disruption leading to vasogenic edema after stroke [65], the use of chloranil would be deleterious as it would result in the breakdown of the BBB. While the detailed analysis of chloranil allows its elimination as a potential drug that could be used in models of brain edema, several other drugs that inhibit both ß-DG and AQP4 clustering in a metalloproteinase-independent manner have been identified in the present study using the high throughput screen on primary astrocyte cultures. Further investigations will determine the effectiveness of these drugs in reducing the clustering of ß-DG and AQP4 at perivascular astrocyte endfeet *in vivo* and in preventing cytotoxic brain edema.
